# Screening of microRNAs for a repressor of hepatitis B virus replication

**DOI:** 10.18632/oncotarget.25557

**Published:** 2018-07-06

**Authors:** Yutaka Naito, Susumu Hamada-Tsutsumi, Yusuke Yamamoto, Akiko Kogure, Yusuke Yoshioka, Koichi Watashi, Takahiro Ochiya, Yasuhito Tanaka

**Affiliations:** ^1^ Division of Molecular and Cellular Medicine, National Cancer Center Research Institute, Tokyo, Japan; ^2^ Department of Virology and Liver Unit, Nagoya City University Graduate School of Medical Sciences, Nagoya, Japan; ^3^ Department of Virology II, National Institute of Infectious Diseases, Tokyo, Japan

**Keywords:** hepatitis B virus, microRNA, miR-204, Rab22a

## Abstract

**Background:**

Hepatitis B virus (HBV) infection is a leading cause of persistent liver diseases, cirrhosis and hepatocellular carcinoma (HCC) worldwide. Since deregulation of microRNA (miRNA) expression by HBV infection contributes to enhanced viral replication and pathogenesis, modulation of miRNA activity can be a novel therapeutic approach towards HBV eradication. As the effects of the vast majority of miRNAs on HBV replication have not been empirically investigated, here, we aim to identify novel therapeutic targets that have a strong antiviral effect on HBV.

**Methods:**

HepG2-hNTCP-C4 cells were infected with HBV, and then were individually transfected with the library mimics of 2048 miRNAs. To assess the amount of intracellular and extracellular DNA and HBsAg, qPCR and ELISA were performed respectively.

**Results:**

From miRNA library screening, we identified 39 miRNAs as candidate repressors of HBV replication. Among them, 9 miRNAs, including miR-204, strongly decreased both HBV DNA and HBsAg in culture supernatant of HepG2-hNTCP-C4 cells. Furthermore, we also showed that inhibition of Rab22a, one of the targets of miR-204, also suppressed intracellular and extracellular HBV DNA expression in HepG2.2.15.7 cells.

**Conclusions:**

Our findings contribute to the understanding of the roles of miRNAs underlying HBV replication and show the possibility of developing a novel strategy for miRNA-mediated HBV treatment.

## INTRODUCTION

The hepatitis B virus (HBV), a small enveloped DNA virus belonging to the Hepadnaviridae family, has a strong preference for hepatocytes and causes acute or chronic liver disease. Approximately 2 billion people have been infected with HBV, more than 240 million people are chronic carriers, and approximately 600,000 patients die each year [1–3]. Chronic HBV infection causes immune-mediate liver damage and oncogenic changes that eventually result in hepatocellular carcinoma (HCC) [4]. Although there are efficient vaccines and treatment strategies for HBV infection, including interferon-based therapy and nucleotide/nucleoside analogues [3, 5], complete eradication of HBV from a patient is still a challenge [3, 5, 6]. Therefore, by finding novel and effective targets of HBV infection and replication, developing better therapeutic strategies that realize improved control of HBV infection was required. Specifically, because the host immune response against HBV infection is exhausted due to circulating high-level virus antigens such as HBsAg, targeting not only HBV replication but also the production/secretion of HBV antigens to reverse an immunosuppression would be an alternative option for the treatment of hepatitis B [7–9].

MicroRNAs (miRNAs) are small non-coding RNAs that post-translationally repress gene expression through sequence-specific base pairing to the 3′ translational region of target gene mRNAs [10]. miRNAs have been reported to participate in many biological processes including cell growth, development, differentiation and homeostasis. Aberrant miRNA expression has been found in a wide range of disorders including viral hepatitis, and several reports have identified that miRNAs control HBV and HCV replication [11–13]. Therefore, miRNAs have been investigated not only to understand mechanisms on disease development but also to exploit a novel class of therapeutic agents for cancer and viral infection. For instance, Miravirsen is a locked nucleic acid (LNA)-modified anti-sense oligonucleotide that targets miR-122, an important host factor of hepatitis C virus replication [13]. Because several kinds of miRNAs including miR-15a, miR-210 and miR-199a-5p have shown an antiviral effect on HBV [14, 15], a miRNA replacement approach as well as an antisense-mediated inhibition of host miRNAs are also feasible. In addition, because miRNAs could suppress viral replication by potentially targeting both host and viral factors by multiple modes of action different from conventional drugs, they could synergistically enhance the antiviral activity when combined with other drugs. Moreover, many miRNAs have been shown to be involved in cancer development and fibrogenesis, suggesting their potential therapeutic advantages for the control of hepatitis B-related liver pathologies such as hepatocellular carcinoma and liver fibrosis [16, 17]. However, most previous studies that sought to find suppressor miRNAs for HBV replication were based on differentially expressed miRNA profiles, and the functional assay was conducted with a limited number of selected miRNA mimics. Therefore, the functional roles of the vast majority of miRNAs in HBV replication are still unclear. In addition, the antiviral effect of miRNAs those are not expressed in hepatocytes with or without HBV infection have also not been assessed.

In the present study, to identify candidate miRNAs as novel therapeutic agents against HBV, we performed a screening using a library containing 2,048 miRNA mimics. From this screening, we identified 39 miRNAs as candidate repressors of HBV replication. Among these, 9 miRNAs strongly suppressed the secretion of both HBV DNA and hepatitis B surface antigen (HBsAg). Furthermore, in the HBV replicon systems, we assessed the antiviral effect of miR-204, which displayed the strongest suppressive effect on HBV replication, and found that inhibition of HBV replication by miR-204 could be, at least in part, mediated by downregulation of Rab22a.

## RESULTS

### Screening of human miRNAs as suppressors of HBV replication

**Figure 1:**
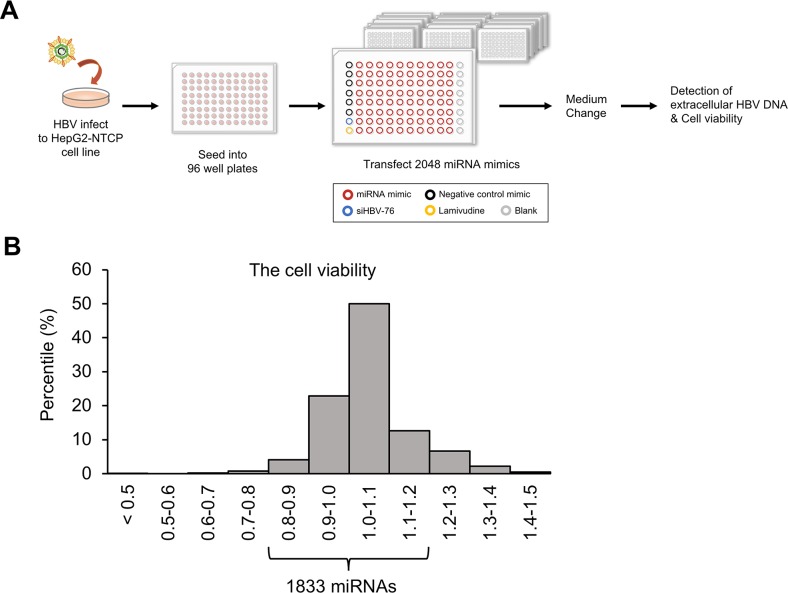
Screening for miRNAs that inhibit HBV replication **(A)** Schematic representation of the screening method. After infection with HBV, we transfected 2,048 human miRNA mimics (red circle) into HepG2-hNTCP-C4 cells, the model of HBV infection. The effect of miRNA mimics was assessed by measuring the amount of extracellular HBV DNA in the culture supernatant. The remaining cells were subjected to MTS assay to exclude miRNAs that influences cell growth and viability. siHBV-76 (blue circle), lamivudine (yellow circle) and six samples for non-specific miRNA mimic (negative control mimic, black circle) were also assessed as positive and negative controls. **(B)** A histogram of the cell viability determined by MTS assay. The x-axis depicts the distribution of cell viability calculated as fold change relative to the negative control mimic-transfected samples. 1,833 miRNAs were selected as their effect on the cell growth rate was less than 20% compared with the negative control mimic.

**Figure 2:**
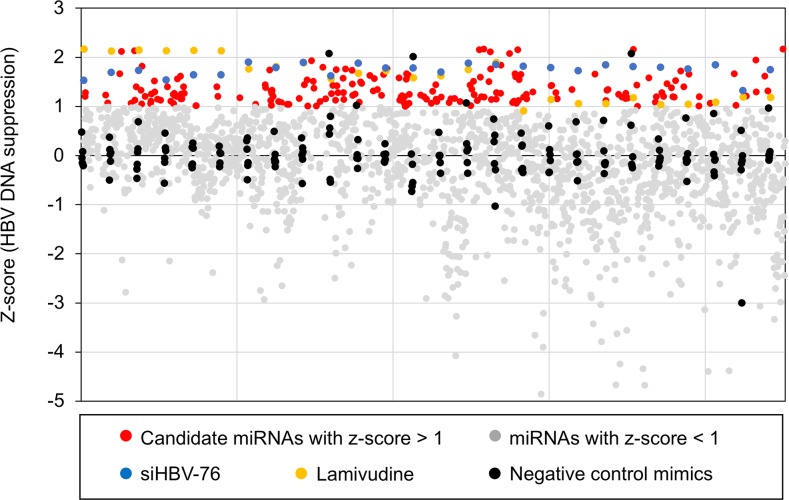
The results of the primary screening Distribution of the Z-scores for each sample calculated from the amount of extracellular HBV DNA. Z-scores corresponding to 1,833 miRNAs are plotted as red dots or grey dots according to cut-off value (Z-score > 1). Z-scores corresponding to Non-specific miRNA mimic, siHBV-76, and lamivudine are plotted as black, blue, and yellow dots, respectively.

**Figure 3:**
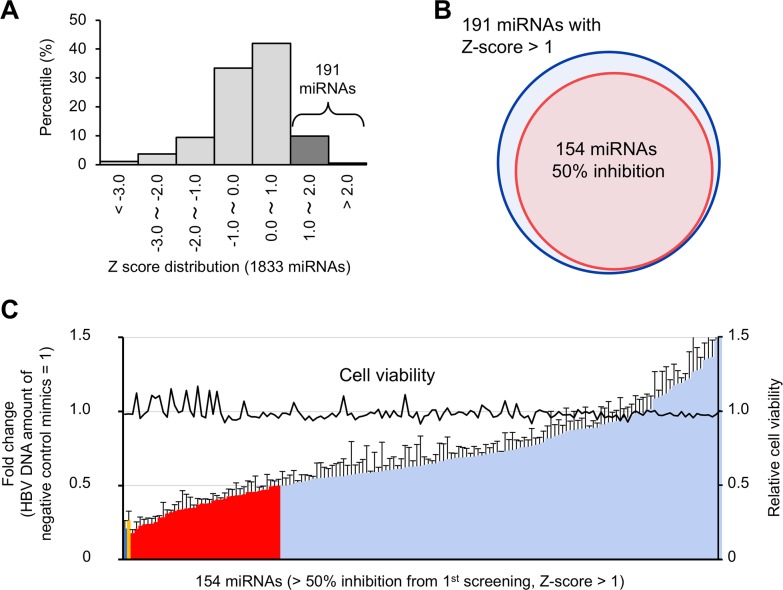
Validation of candidate antiviral miRNAs **(A)** A histogram of the Z-score for 1833 miRNAs, indicating 191 candidate antiviral miRNAs with Z-scores > 1. **(B)** One hundred and fifty-four miRNAs that decreased extracellular HBV DNA more than 50% were included in the 191 candidate miRNAs with Z-scores > 1. **(C)** The antiviral activity of these 154 miRNAs was validated in triplicate. The amount of extracellular HBV DNA is depicted as fold change relative to negative control mimic-treated samples. The red bars indicate 39 miRNAs that decrease the amount of extracellular HBV DNA more than 50%. The blue and yellow bar shows the amounts of HBV DNA in the siHBV-76- and Lamivudine-treated samples, respectively. Error bars represent the s.d.

To identify miRNAs that affect HBV replication, we performed a high-throughput screening using a library of miRNA mimics (2,048 human mature miRNAs). A HepG2-hNTCP-C4 cell line was used for the experiment because it possesses stable susceptibility to HBV infection [18, 19]. As shown in Figure 1A, HepG2-hNTCP-C4 cells were infected with HBV and then individually transfected with each miRNA mimic. The amount of extracellular HBV DNA in the culture supernatant was quantified by qPCR. HBV siRNA (siHBV-76) and lamivudine were used as positive controls, and a non-specific miRNA mimic was also used as a negative control (negative control mimic) in each 96-well plate (Figure 1A). To exclude miRNAs that influence the cell growth and viability, we performed MTS assay and selected 1,833 miRNAs, as their effect on the cell growth were less than 20% compared to the negative control mimic (Figure 1B). Using a cut off value of Z-score > 1, 191 miRNAs with a negative effect on the level of extracellular HBV DNA were selected as candidate suppressors of HBV replication (Figure 2 and Figure 3A, and Supplementary Table 1). Interestingly, we also identified 260 miRNAs that increased the extracellular HBV DNA (Z-score < 1, Figure 2 and Supplementary Table 2). As expected, siHBV-76 and lamivudine (except one sample) significantly decreased the amount of extracellular HBV DNA (Z-score > 1, Figure 2). The negative control mimic has basically no impact on the HBV replication (− 1 < Z-score < 1, Figure 2).

### Validation of antiviral miRNA candidates

Among the 191 miRNAs with a negative effect on HBV replication, 154 miRNAs reduced extracellular HBV DNA more than 50% compared to the negative control mimic (Figure 3A and 3B). To evaluate reproducibility, these 154 miRNAs were further tested by secondary validation screening performed in triplicate. Finally, 39 miRNAs were selected as suppressors of HBV replication that significantly decreased the amount of extracellular HBV DNA more than 50% (Figure 3C). Cell growth assay confirmed that these 39 miRNAs did not affect the viability of HepG2-hNTCP-C4 cells (Figure 3C). siHBV-76 and lamivudine also showed strong antiviral activity (Figure 3C). These data suggest that our screening approaches successfully identified antiviral miRNAs against HBV replication.

### Identification of miR-204 as a regulator of HBV replication

**Figure 4:**
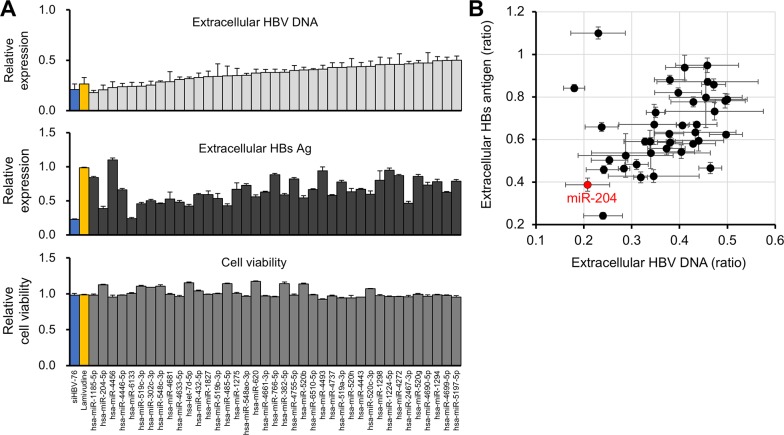
Identification of miR-204 as a candidate miRNA for HBV suppression **(A)** The effect of 39 miRNAs, siHBV-76 and Lamivudine on the extracellular HBV DNA level (top) and cell viability (bottom) in the second validation screening. The effect of these 39 miRNAs, siHBV-76 and Lamivudine on the extracellular HBsAg level was assessed (middle). The values are depicted as fold change relative to the extracellular HBV DNA level, extracellular HBsAg and cell viability of the non-specific miRNA mimic-treated group. The assay was carried out in triplicate. Error bars represent the s.d. **(B)** The scatter plot of the extracellular HBV DNA level and extracellular HBsAg level (the ratio compared with negative control mimics) of 39 miRNA mimic-transfected samples, showing that miR-204 strongly suppressed the secretion of both extracellular HBV DNA and HBsAg in HepG2-hNTCP-C4 cells.

To investigate the antiviral effects of the candidate 39 miRNAs on HBV replication more in detail, we measured extracellular HBsAg levels in the supernatant (Figure 4A middle) and the results were aligned with the validation screening data of the level of extracellular HBV DNA and growth rate (Figure 4A top and bottom, respectively). Interestingly, 9 miRNAs suppressed the secretion of both HBV DNA and HBsAg more than 50% (Figure 4A and 4B). Notably, miR-204 displayed the strongest antiviral effect on both HBV replication and HBsAg secretion among these candidates (Figure 4A and 4B). siHBV-76 and lamivudine also suppressed HBV replication (Figure 4A). However, lamivudine failed to inhibit the secretion of HBsAg because its mode of action is the inhibition of reverse transcription [20]. These data suggest that 9 miRNAs, in particular miR-204, are potent inhibitors of HBV replication.

We next investigated the expression profiles of the selected 9 miRNAs in the normal livers by searching for a public database for miRNA abundance in various tissue biopsies including liver [21]. From this database, we found 8 of 9 candidate miRNA expressions in the liver (Supplementary Figure 1A). The remaining candidate miRNA, miR-519-3p has been reported to be expressed in the placental trophoblast cells [22]. Next, we investigated the expression profiles of these candidate miRNAs in 20 different human tissues. As shown in Supplementary Figure 1B, 8 candidate miRNAs broadly expressed in various organs and were not preferentially distributed in the liver. To elucidate the functional role of these miRNAs, target prediction for these 9 miRNAs was also carried out using TargetScan (Supplementary Table 3). Gene ontology analysis showed that these predicted target genes were involved in several molecular functions such as “transcription” and “DNA binding” (Supplementary Figure 2). Therefore, it suggests that some of these 9 miRNAs might exert the antiviral effect by targeting DNA binding- or transcription- related genes.

### miR-204 suppressed HBV replication in a HepG2.2.15.7 cell line through targeting Rab22a

**Figure 5:**
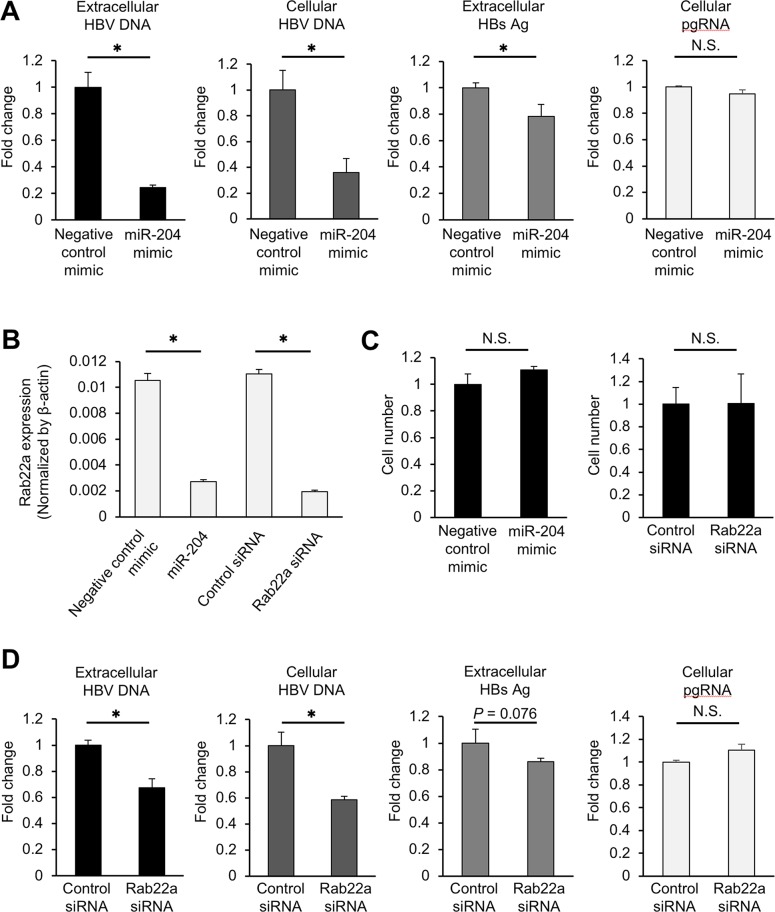
miR-204 and Rab22a suppress viral replication in HepG2.2.15.7 cells **(A)** The effect of miR-204 on the levels of extracellular HBV DNA, intracellular HBV DNA, extracellular HBsAg and pgRNA in HepG2.2.15.7 cells. **(B)** The effect of miR-204 and Rab22a siRNA on the expression of Rab22a in HepG2.2.15.7 cells. **(C)** The effect of miR-204 and Rab22a siRNA on cell growth rate of HepG2.2.15.7 cells. **(D)** The effect of Rab22a siRNA on the levels of extracellular HBV DNA, intracellular HBV DNA, extracellular HBsAg and pgRNA in HepG2.2.15.7 cells. The values are depicted as fold change relative to non-specific miRNA mimic (negative control mimic) or negative control siRNA (control siRNA). Error bars represent the s.d. deduced by Student's *t*-test (^*^P < 0.05). N.S., no significant difference. The data are representative of at least three independent experiments.

Given the result of the miRNA library screening showing that miR-204 was a potent suppressor of HBV replication in a viral infection model, we next sought to assess the effect of miR-204 mimic using an HBV replicon system [19]. The HepG2.2.15.7 cell line is a strong HBV producer subcloned from the HepG2.2.15 cell line, which had been established by introducing the HBV genome into the chromosome and is used as the model for HBV replication [23–25]. We selected this cell line because it robustly produces HBV particles and HBsAg compared with HepG2-hNTCP-C4 cells. The cells were transfected with miR-204 mimic or negative control mimic, and the levels of HBV DNA and HBsAg were monitored. Compared with the control mimic, the miR-204 mimic significantly reduced extracellular HBV DNA, intracellular HBV DNA and extracellular HBsAg (Figure 5A). These data suggest that miR-204 is able to suppress HBV replication both in infection and replicon systems [19]. On the other hand, miR-204 did not influence the expression of pregenomic RNA (pgRNA) (Figure 5A), indicating that miR-204 act on the posttranscriptional steps. To investigate the roles of miR-204 target genes in HBV replication, we focused on Rab22a which had the highest prediction score among the candidates of miR-204 target genes (Supplementary Table 3). In a previous report, Rab22a, a member of the RAS oncogene family and one of the endosome-associated proteins, was experimentally validated as a target of miR-204 using colorectal cancer cells [26]. Corresponding to this report, we also confirmed that Rab22a expression was significantly suppressed in miR-204-transfected HepG2.2.15.7 cells (Figure 5B). Several lines of evidence have indicated that HBV infection and replication take place in the endocytic compartment and are modulated by RAB family member proteins [27, 28]. To assess the impact of Rab22a on HBV replication, HepG2.2.15.7 cells were transfected with a Rab22a-specific small interfering RNA (siRNA) or a non-specific siRNA. Rab22a expression was significantly decreased by Rab22a siRNA treatment (Figure 5B). Neither treatment with miR-204 mimic nor Rab22a siRNA affected the viability of HepG2.2.15.7 cells (Figure 5C). Interestingly, the amount of both the extracellular and intracellular HBV DNA was significantly decreased in the cells transfected with Rab22a siRNA compared with the control (Figure 5D). However, although Rab22a siRNA treatment tended to decrease extracellular HBsAg, this reduction was not statistically significant (Figure 5D). In addition, Rab22a siRNA did not suppress pgRNA expression (Figure 5D). We then examined if the upregulation of Rab22a further increase HBV replication and HBsAg secretion by transfecting Hep G2.2.15.7 cells with a human Rab22a expression vector. As shown in Supplementary Figure 3, forced expression of Rab22a did not affect viral replication. Taken together, our results demonstrated that miR-204 is a potent suppressor of HBV replication, and suggested that the reduction of Rab22a expression might partially account for the antiviral effect of miR-204.

## DISCUSSION

Antiviral agents approved for the treatment of chronic HBV infection can be divided into two major classes: interferon and nucleotide/nucleoside analogues. Although interferon-based therapy has advantages due to its finite duration of therapy without the development of drug resistance, the direct antiviral effect of interferon on HBV infection might be relatively weak, according to previous studies [5, 29, 30]. In contrast, the nucleotide/nucleoside analogues directly target the viral polymerase and are highly effective at inhibiting HBV replication. However, nucleotide/nucleoside analogues have a risk for developing viral mutations associated with HBV drug resistance [5]. Therefore, these treatment approaches remain unsatisfactory, and a novel therapeutic approach for the eradication of viruses from patients with chronic HBV infection is still needed. In the present study, we focused on miRNA mimics as novel therapeutic agents for HBV infection. Because miRNAs influence viral replication through directly or indirectly targeting viral genes [12, 15], miRNA mimics might possess suppressive effects on HBV replication through targeting multiple pathways in host cells regardless of the mutation status of HBV. Through the functional screening of a miRNA library, we identified several antiviral miRNAs, one of which was miR-204, which has been reported as a suppressor of HBV replication, without targeting viral genes [4]. The restoration of compromised HBV-specific immunity is critical to eliminate cccDNA-containing hepatocytes and to establish life-long immune control of HBV. Our findings that the potent 9 miRNAs significantly suppressed extracellular HBsAg as well as viral DNA level, suggesting their clinical potential to reduce circulating HBsAg which hamper immune response against HBV. Thus, these identified HBV-suppressive miRNAs could be applicable for a novel therapeutic approach to overcome the obstacles of the conventional HBV treatment.

Our results showed that miR-204 mimic displayed the strongest suppressive effect on the amount of HBV DNA and HBsAg among 9 candidate miRNAs. Consistent with our findings, Huang et al. recently reported a suppressive effect of miR-204 on HBV replication. They suggested that miR-204 might interfere with HBV capsid assembly by targeting HBc antigen expression without a direct interaction with HBV-specific RNAs [4]. They also found that HBV might suppress miR-204 expression through STAT3 signaling pathway [4]. Based on our findings, because Rab22a is at least partly associated with the miR-204-mediated suppression of HBV replication, several pathways in host cells seem to be governed by miR-204 for the defense against HBV replication. As shown by the results from the library screening and validation assay, miR-204 mimic has no effect on cell growth; however, we could not exclude the possibility that administration of abundant miR-204 is harmful to normal hepatocytes, as miR-204 possesses a strong antiviral activity by affecting multiple pathways in the host cells. The understanding of the detailed physiological molecular function of miR-204 is required to clarify the side effects of miR-204 in normal hepatocytes before considering it as a therapeutic target.

We also revealed that down-regulation of Rab22a expression by miR-204 significantly suppressed the amount of extracellular and intracellular HBV DNA in HepG2.2.15.7 cells. Rab22a is a member of the Rab GTPase family, which includes more than 60 members and is involved in multiple membrane trafficking pathways such as the endocytic pathway [31]. Like other Rab proteins, Rab22a also contributes to the endocytic pathway through transportation of the endocytosed cargo to the endocytic recycling compartment [32–34]. Recently, a number of reports have demonstrated that Rab-mediated endosomal trafficking is closely associated with the establishment of HBV infection, viral particle transport and secretion [27, 28]. However, to date, the roles of Rab22a in the HBV life cycle have not been elucidated. Therefore, our data suggest, for the first time, that Rab22a contributes to the HBV production and replication machinery. Forced expression of Rab22a did not affect the amount of HBV DNA, suggesting that endogenous level of Rab22a expression is sufficient for the HBV replication. While siRab22a treatment decreased the expression of Rab22a as efficiently as miR-204, its suppressive effect against HBV replication was smaller than miR-204. Therefore, downregulation of miR-204 target genes other than Rab22a may also contribute to its antiviral activity. The precise mechanism by which Rab22a and the endocytic recycling pathway mediate the HBV lifecycle should be investigated in a future study.

In the present study, we focused on the miRNA mimics with a negative effect on HBV replication (decreasing extracellular HBV DNA) in the primary screening. However, our screening results also identified over 200 miRNA mimics with positive effects on HBV replication (increasing extracellular HBV DNA), although we have not carried out the validation study for them yet. Previous reports have shown that several miRNAs, including miR-1 [35], miR-372/373 [36], and miR-501 [37], promote HBV replication through targeting host gene expression. For instance, miR-501 promoted HBV replication in HBV-producing cell lines through targeting HBXIP, an inhibitor of viral protein [37]. Notably, our screening methods also identified miR-501 as a positive regulator of HBV replication (Z-score < −1, Supplementary Table 2). Thus, suppression of miRNAs with positive effects on HBV replication by introducing anti-sense nucleic acids might be another choice for a therapeutic strategy.

Because of unavoidable technical errors in the extraction and quantification of HBV DNA, our primary screening would have selected several false positives as HBV suppressors or have overlooked some microRNAs with marginal antiviral activity. However, by further validating the candidate 154 miRNAs in triplicate, we successfully identified 39 miRNAs that effectively decreased HBV DNA in the supernatant with high statistically significance (data not shown). Several miRNAs that were previously reported to have antiviral activity against HBV replication such as miR-141 [38], 210 [15], 939 [11] and 1236 [4] were not selected in our system. This inconsistency may be due to the difference in the experimental settings each study used, including HBV replication model, timing and duration of microRNA treatment, and assay readouts (extracellular HBV DNA/HBsAg, or intracellular HBV DNA). Despite these issues, our results demonstrating the identification of 9 miRNAs including miR-204 that greatly suppress both HBV DNA and HBsAg secretion, supports the validity of our screening system using *in vitro* model that recapitulate entire HBV replication cycle.

In conclusion, we identified several potent suppressors of HBV replication by comprehensive screening of human miRNA library. In particular, among these candidate miRNAs, miR-204 might be a useful target for miRNA-based therapy for HBV infection. Although further studies are required to clarify the antiviral activity and unintended side-effect of these candidate miRNAs *in vivo*, our findings will provide great advantages for developing novel therapeutics and understanding the biological processes underlying HBV replication.

## MATERIALS AND METHODS

### Cell culture

HepG2-hNTCP-C4 cells [39] were maintained at 37°C in a humidified 5% CO2 incubator in Dulbecco's modified Eagle's medium (DMEM) (Gibco, Foster, CA, USA) that contained 10% foetal bovine serum, 100 units/mL of penicillin and 100 mg/mL of streptomycin. HepG2.2.15.7 cells [23] were maintained at 37°C in a humidified 5% CO2 incubator in DMEM/Nutrient Mixture F-12 with Glutamax (DMEM/F12 + Glutamax) (Gibco) that contained 10% foetal bovine serum, 100 units/mL of penicillin and 100 mg/mL of streptomycin, 10 mM HEPES (Sigma-Aldrich, St. Louis, MO, USA), 5 μg/mL insulin (Wako, Tokyo, Japan), 50 μM hydrocortisone (Sigma-Aldrich) and 400 μg/mL G418.

### MicroRNA library screening

High-throughput miRNA screening was performed using the Human miRIDIAN miRNA Mimic Library constructed based on miRBase v19.0 (GE Healthcare, Piscataway, NJ, USA). HepG2-hNTCP-C4 cells were inoculated with HBV derived from HepG2.2.15 cells at 3,000 genomes per cell in the presence of 3% DMSO and 4% polyethylene glycol 8000 (Sigma-Aldrich) as previously described [39]. Two days after infection (day 2), the cells were harvested and plated on collagen-coated 96-well multiplates at a density of 2.5 × 10^4^ cells/well in the presence of 0.5% DMSO. We allocated 80 wells for test miRNA mimics, six wells for non-specific miRNA mimic (negative control mimic), one well each for siHBV-76 and lamivudine in each 96-well plate. In total, we used 26 plates to screen 2,048 miRNA mimics. On day 4, the cells were then transfected with miRNAs using Lipofectamine RNAiMAX (Thermo Fisher Scientific, Waltham, MA, USA) according to the manufacturer's instructions. Specifically, 1.5 pmol of each miRNA was diluted with 5 μL of Opti-MEM I reduced serum medium (Thermo Fisher Scientific) and mixed with 0.3 μL of RNAiMAX also diluted with 5 μL of Opti-MEM I. After incubation at room temperature for 5 minutes, the mixture was added to the cells. As positive controls, 1.5 pmol of siHBV-76 [40] diluted with 5 μL of Opti-MEM I and 5 μL of 400 nM lamivudine were mixed with RNAiMAX and added to the cells. The final concentration of lamivudine was 20 nM. On day 5, the medium was removed and replenished with fresh medium containing 0.5% DMSO. The culture supernatants were collected on day 9 and tested for the HBV DNA level as previously described [41]. To measure viability, we performed MTS assay as follows. The remaining cells were incubated with 20-fold diluted CellTiter 96 Aqueous One Solution Reagent (Promega, Fitchburg, WI, USA) at 37°C for 4 hours. The cell viability was calculated according to the manufacturer's instructions. From the result of the primary screening (n = 1), we identified 191 miRNAs with a negative effect on extracellular HBV DNA replication in HepG2-hNTCP-C4 cell. Among these miRNAs, we selected 154 miRNAs that suppressed extracellular HBV DNA more than 50% compared to the negative control mimic. A secondary validation screening was performed by using 154 miRNA mimics (n = 3).

### HBV replication analysis

For quantification of HBV DNA, total DNA was extracted from the culture media of HepG2.2.15.7 cells using a QIAamp DNA Mini Kit (Qiagen, Hilden, Germany). Extracellular HBV DNA was quantified by real-time quantitative PCR using StepOne Plus and TaqMan Universal PCR Master Mix (Thermo Fisher Scientific). HBV DNA was amplified using primers HBV-F (5′-CACATCAGGATTCCTAGGACC-3′) and HBV-R (5′-AGGTTGGTGAGTGATTGGAG-3′) and TaqMan probe HBV-FT (5′-FAM-CAGAGTCTA GACTCGTGGTGGACTTC-TAMRA-3′). The extracellular HBsAg was quantified by Enzygnost^®^ HBsAg 6.0 (SIEMENS, Marburg, Germany). PgRNA was quantified by reverse transcription quantitative PCR as previously described [32].

### Transfection

Transfection of miRNAs into the cells was performed with Lipofectamine RNAiMAX reagent (Thermo Fisher Scientific) according to the manufacturer's instructions. Briefly, the cells were seeded at 50 - 60% confluence the day before transfection. The miR-204 mimic or negative control miRNA (Ambion, Austin, TX, USA) was used at a final concentration of 50 nM to investigate the effect of miR-204 on HBV replication. Rab22a siRNA or ALL STAR negative control mimic (Thermo Fisher Scientific) was used at a final concentration of 50 nM to investigate the effect of Rab22a on HBV replication. Five days after transfection, total RNA was extracted using miRNeasy Mini Kit (Qiagen) according to the manufacturer's instructions, and Rab22a expression was then determined by qPCR. Total DNA was extracted from the cells or culture supernatant using QIAamp DNA Mini Kit (Qiagen), and the amount of HBV DNA was determined by qPCR. Transfection of a CMV-driven human Rab22a expression vector (EX-W1322-M02, GeneCopoeia) and control vector (EX-NEG-M02, GeneCopoeia) into the cells was performed with Lipofectamin 3000 reagent (Thermo Fisher Scientific) according to the manufacturer's instructions. Briefly, the cells were seeded at 50 - 60% confluence the day before transfection. The Rab22a expression vector or control vector were used at a final concentration of 5 μg to investigate the effect of Rab22a expression on HBV replication, as described in Supplementary Figure 3.

### DAVID gene ontology

The representation of Gene Ontology (GO) molecular function terms within the lists of the predicted miRNA target genes by TargetScan v7.1 (http://www.targetscan.org/vert_71/) was investigated using the Database for Annotation, Visualization, and Integrated Discovery (DAVID; http://david.abcc.ncifcrf.gov). The terms were ranked by –Log10 (*p*-value), as described in Supplementary Figure 2.

### Quantitative RT-PCR (qRT-PCR)

Quantification of Rab22a and β-actin mRNA was performed using real-time fluorescence detection as described previously [42]. Rab22a primer sequences are as follows: 5′-GAACGATTTCGTGCCTTAGC-3′ and 5′-GCTGTCGAAGCTCTTTCACC-3′. β-actin primer sequences are as follows: 5′-TCACCGAGCGCGGCT-3′ and 5′-TAATGTCACGCACGATTTCCC-3′. qRT-PCR was performed with a Platinum SYBR Green qPCR SuperMix-UDG (Thermo Fisher Scientific). For analysis of miR-204 and U44 expression levels were performed using TaqMan assays (Thermo Fisher Scientific). Total RNA was extracted with a miRNeasy Mini Kit (Qiagen) according to the manufacturer's instructions. Expression levels of Rab22a and miR-204 were normalized by β-actin and RNU6B expression respectively and calculated using the ΔΔCt method.

### Statistical analysis

In the primary miRNA screening, we applied Z-score statistics for the summation of the normalized extracellular HBV DNA amount. To provide the reduction of extracellular HBV DNA amount, we calculate Z-score as following formula. (Z-score = 0 - [observed value – median value of the reference population] / standard derivation value of the reference population). In other data, statistical analysis was performed using Student's *t*-test. Values are presented as the mean ± standard deviation (SD) of at least three independent experiments.

## SUPPLEMENTARY MATERIALS FIGURES AND TABLES








