# Supplementary material for: Screening of microRNAs for a repressor of hepatitis B virus replication
Source: Oncotarget. 2018 Jul 6;9(52):29857–68. doi: 10.18632/oncotarget.25557 (PMC6057454; doi:10.18632/oncotarget.25557)
Supplement: Supplementary file 1 [file oncotarget-09-29857-s001.pdf]

Screening of microRNAs for a repressor of hepatitis B virus replication

SUPPLEMENTARY MATERIALS

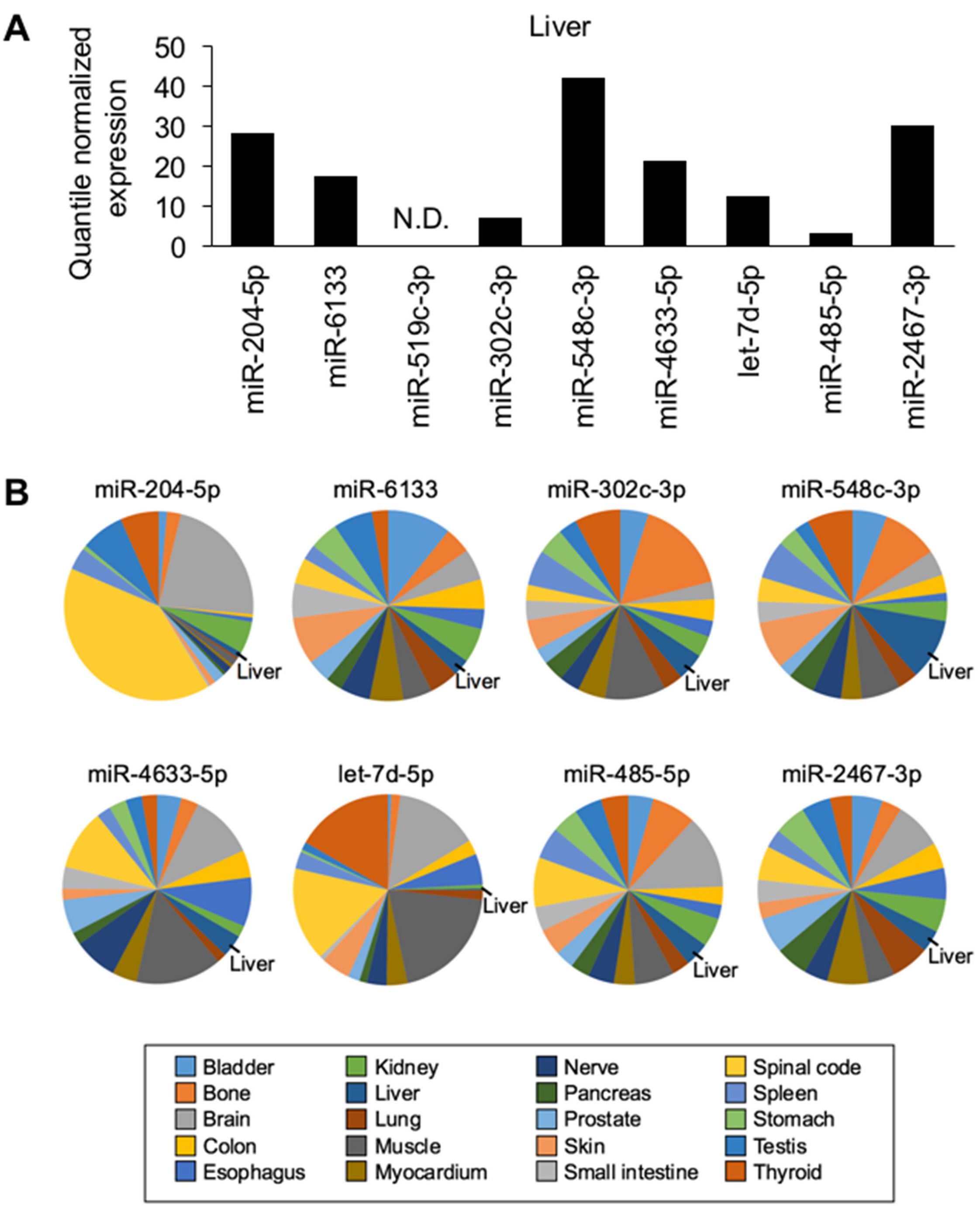

Supplementary Figure 1: Expression of 9 antiviral miRNAs in normal human tissues. (A) Quantile normalized expression of 9 miRNAs in liver. There data were assessed using the Database for Human miRNA tissue atlas (https://ccb-web.cs.uni- saarland.de/tissueatlas/). **(B)** The expression of 8 candidate miRNAs in 20 human tissues including liver.

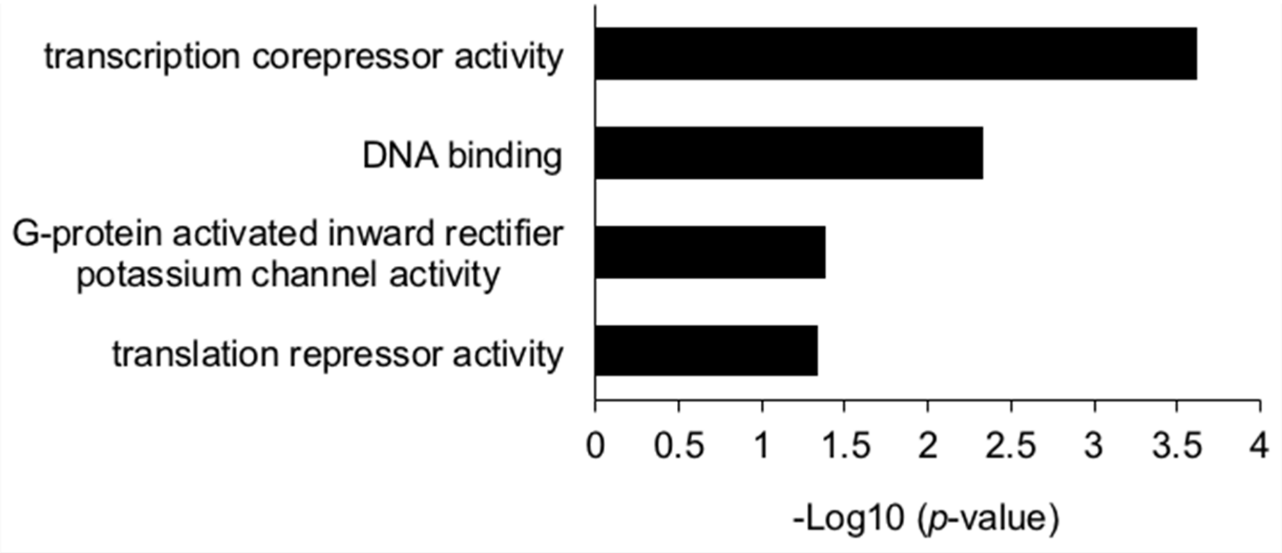

Supplementary Figure 2: Gene Ontology (GO) enrichment analysis of the predicted target genes of 9 antiviral miRNAs. Top four most significant GO molecular function.

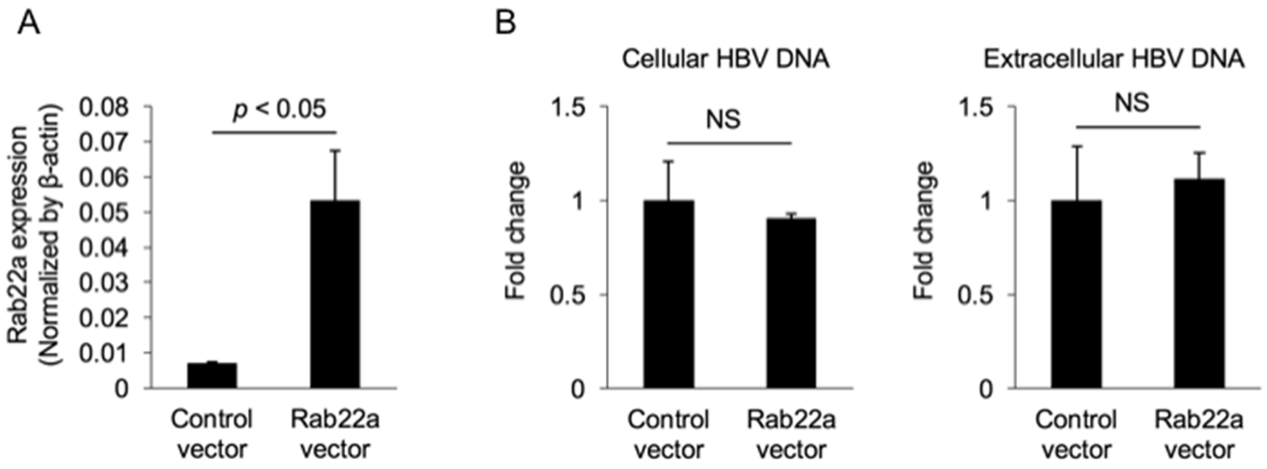

Supplementary Figure 3: The effect of Rab22a expression on the amount of cellular and extracellular HBV DNA in HepG2.2.15.7 cells. (A) Relative Rab22a expression levels in HepG2.2.15.7 cells with Rab22a expression vector (Rab22a vector) and control vector. The values on the y-axis are depicted relative to Rab22a expression of the control vector transfected group. **(B)** Forced expression of Rab22a in HepG2.2.15.7 cells (Rab22a vector) did not affect the amount of cellular HBV DNA and extracellular HBV DNA. The values on the y-axis are depicted relative to the amount of cellular HBV DNA and extracellular HBV DNA of the negative control vector transfected group (Control vector). Error bars represent the s.d. Student's *t*-test. NS, no significant difference.

Supplementary Table 1: miRNAs with negative effect of HBV replication in initial screening.

See Supplementary File 1

Supplementary Table 2: miRNAs with positive effect of HBV replication in initial screening.

See Supplementary File 2

Supplementary Table 3: Putative target genes of 9 candidate miRNAs.

See Supplementary File 3
