# Supplementary material for: Screening of microRNAs for a repressor of hepatitis B virus replication
Source: Oncotarget. 2018 Jul 6;9(52):29857–68. doi: 10.18632/oncotarget.25557 (PMC6057454; doi:10.18632/oncotarget.25557)
Supplement: Supplementary file 2 [file oncotarget-09-29857-s002.docx]

**Supplementary Table 1: miRNAs with negative effect of HBV replication in initial screening**

|  | **miRNA** | **miRNA mimic ID of library** | **Reverse_z-score** |  |
| --- | --- | --- | --- | --- |
|  | **hsa-miR-3184-3p** | **2248** | **2.18** |  |
|  | **hsa-miR-4258** | **1290** | **2.17** |  |
|  | **hsa-miR-203b-3p** | **1769** | **2.17** |  |
|  | **hsa-miR-4312** | **1275** | **2.16** |  |
|  | **hsa-miR-1292-3p** | **1398** | **2.16** |  |
|  | **hsa-miR-362-5p** | **170** | **2.14** |  |
|  | **hsa-miR-191-5p** | **129** | **2.12** |  |
|  | **hsa-miR-4263** | **1310** | **2.12** |  |
|  | **hsa-miR-365b-5p** | **1372** | **2.09** |  |
|  | **hsa-miR-6133** | **2161** | **1.95** |  |
|  | **hsa-miR-876-3p** | **738** | **1.94** |  |
|  | **hsa-miR-4330** | **1344** | **1.84** |  |
|  | **hsa-miR-374a-5p** | **194** | **1.83** |  |
|  | **hsa-miR-1224-5p** | **774** | **1.81** |  |
|  | **hsa-miR-3605-5p** | **980** | **1.78** |  |
|  | **hsa-miR-4270** | **1312** | **1.77** |  |
|  | **hsa-miR-1294** | **846** | **1.77** |  |
|  | **hsa-miR-1185-5p** | **817** | **1.76** |  |
|  | **hsa-miR-483-5p** | **604** | **1.75** |  |
|  | **hsa-miR-1253** | **867** | **1.74** |  |
|  | **hsa-miR-4795-3p** | **1920** | **1.72** |  |
|  | **hsa-miR-142-3p** | **123** | **1.68** |  |
|  | **hsa-miR-5681b** | **2092** | **1.67** |  |
|  | **hsa-miR-506-5p** | **1390** | **1.67** |  |
|  | **hsa-miR-3679-5p** | **1042** | **1.67** |  |
|  | **hsa-miR-1273f** | **1947** | **1.67** |  |
|  | **hsa-miR-4633-5p** | **1646** | **1.66** |  |
|  | **hsa-miR-2115-3p** | **968** | **1.66** |  |
|  | **hsa-miR-548ao-3p** | **1408** | **1.66** |  |
|  | **hsa-miR-3682-3p** | **1048** | **1.64** |  |
|  | **hsa-miR-1827** | **925** | **1.64** |  |
|  | **hsa-miR-520d-3p** | **318** | **1.62** |  |
|  | **hsa-miR-6510-5p** | **2190** | **1.62** |  |
|  | **hsa-miR-381-5p** | **1392** | **1.61** |  |
|  | **hsa-miR-4316** | **1278** | **1.60** |  |
|  | **hsa-miR-3659** | **1018** | **1.59** |  |
|  | **hsa-miR-4737** | **1818** | **1.59** |  |
|  | **hsa-miR-1286** | **839** | **1.58** |  |
|  | **hsa-miR-1911-3p** | **939** | **1.58** |  |
|  | **hsa-miR-520c-3p** | **308** | **1.56** |  |
|  | **hsa-miR-3678-3p** | **1041** | **1.56** |  |
|  | **hsa-miR-766-5p** | **1403** | **1.56** |  |
|  | **hsa-miR-892a** | **723** | **1.55** |  |
|  | **hsa-miR-2467-3p** | **1904** | **1.53** |  |
|  | **hsa-miR-448** | **234** | **1.53** |  |
|  | **hsa-miR-744-3p** | **743** | **1.52** |  |
|  | **hsa-miR-4296** | **1256** | **1.52** |  |
|  | **hsa-miR-2355-3p** | **1306** | **1.51** |  |
|  | **hsa-miR-1272** | **895** | **1.50** |  |
|  | **hsa-miR-1275** | **898** | **1.48** |  |
|  | **hsa-miR-4770** | **1877** | **1.47** |  |
|  | **hsa-miR-1254** | **868** | **1.46** |  |
|  | **hsa-miR-431-3p** | **589** | **1.45** |  |
|  | **hsa-miR-93-3p** | **715** | **1.45** |  |
|  | **hsa-miR-3125** | **1136** | **1.45** |  |
|  | **hsa-miR-4715-5p** | **1780** | **1.44** |  |
|  | **hsa-miR-3198** | **1246** | **1.44** |  |
|  | **hsa-miR-4446-5p** | **1507** | **1.44** |  |
|  | **hsa-miR-205-3p** | **806** | **1.43** |  |
|  | **hsa-miR-4443** | **1505** | **1.43** |  |
|  | **hsa-miR-1298** | **819** | **1.43** |  |
|  | **hsa-miR-548c-3p** | **434** | **1.41** |  |
|  | **hsa-miR-4800-5p** | **1930** | **1.41** |  |
|  | **hsa-miR-302c-3p** | **176** | **1.41** |  |
|  | **hsa-miR-520h** | **328** | **1.41** |  |
|  | **hsa-miR-432-5p** | **276** | **1.41** |  |
|  | **hsa-miR-491-3p** | **590** | **1.39** |  |
|  | **hsa-miR-4779** | **1891** | **1.38** |  |
|  | **hsa-miR-885-5p** | **745** | **1.38** |  |
|  | **hsa-miR-514b-5p** | **1251** | **1.36** |  |
|  | **hsa-miR-1973** | **949** | **1.35** |  |
|  | **hsa-miR-4661-3p** | **1688** | **1.35** |  |
|  | **hsa-miR-4493** | **1572** | **1.35** |  |
|  | **hsa-miR-4658** | **1684** | **1.34** |  |
|  | **hsa-miR-372** | **191** | **1.34** |  |
|  | **hsa-miR-576-3p** | **611** | **1.34** |  |
|  | **hsa-miR-6515-5p** | **2200** | **1.33** |  |
|  | **hsa-miR-1909-3p** | **935** | **1.32** |  |
|  | **hsa-miR-556-3p** | **608** | **1.32** |  |
|  | **hsa-miR-3116** | **1126** | **1.32** |  |
|  | **hsa-miR-6134** | **2162** | **1.31** |  |
|  | **hsa-miR-642b-5p** | **1447** | **1.30** |  |
|  | **hsa-miR-429** | **235** | **1.30** |  |
|  | **hsa-miR-4278** | **1330** | **1.29** |  |
|  | **hsa-miR-1180** | **822** | **1.29** |  |
|  | **hsa-miR-1287** | **840** | **1.29** |  |
|  | **hsa-let-7e-5p** | **13** | **1.29** |  |
|  | **hsa-miR-4760-5p** | **1860** | **1.29** |  |
|  | **hsa-miR-4456** | **1520** | **1.29** |  |
|  | **hsa-miR-4329** | **1343** | **1.28** |  |
|  | **hsa-miR-519a-3p** | **330** | **1.28** |  |
|  | **hsa-miR-526b-3p** | **298** | **1.28** |  |
|  | **hsa-miR-4699-5p** | **1745** | **1.27** |  |
|  | **hsa-miR-574-5p** | **610** | **1.27** |  |
|  | **hsa-miR-513c-5p** | **801** | **1.27** |  |
|  | **hsa-miR-345-5p** | **224** | **1.26** |  |
|  | **hsa-miR-520g** | **320** | **1.26** |  |
|  | **hsa-miR-1263** | **877** | **1.26** |  |
|  | **hsa-miR-519c-3p** | **294** | **1.26** |  |
|  | **hsa-miR-3685** | **1051** | **1.26** |  |
|  | **hsa-miR-4681** | **1721** | **1.25** |  |
|  | **hsa-miR-5197-5p** | **1426** | **1.25** |  |
|  | **hsa-miR-4293** | **1259** | **1.25** |  |
|  | **hsa-miR-204-5p** | **78** | **1.24** |  |
|  | **hsa-miR-3150b-3p** | **1087** | **1.24** |  |
|  | **hsa-miR-519b-3p** | **299** | **1.24** |  |
|  | **hsa-miR-449c-5p** | **962** | **1.23** |  |
|  | **hsa-miR-1256** | **870** | **1.23** |  |
|  | **hsa-miR-3667-5p** | **1028** | **1.22** |  |
|  | **hsa-miR-6071** | **2131** | **1.22** |  |
|  | **hsa-let-7d-5p** | **12** | **1.21** |  |
|  | **hsa-miR-382-5p** | **204** | **1.21** |  |
|  | **hsa-miR-620** | **438** | **1.21** |  |
|  | **hsa-miR-4763-3p** | **1866** | **1.21** |  |
|  | **hsa-miR-550a-5p** | **615** | **1.21** |  |
|  | **hsa-miR-520b** | **305** | **1.21** |  |
|  | **hsa-miR-3665** | **1025** | **1.21** |  |
|  | **hsa-miR-3928** | **1098** | **1.21** |  |
|  | **hsa-miR-4782-3p** | **1895** | **1.20** |  |
|  | **hsa-miR-4320** | **1282** | **1.20** |  |
|  | **hsa-miR-4755-5p** | **1842** | **1.20** |  |
|  | **hsa-miR-5004-5p** | **1980** | **1.20** |  |
|  | **hsa-miR-3674** | **1035** | **1.20** |  |
|  | **hsa-miR-548ao-5p** | **1417** | **1.20** |  |
|  | **hsa-miR-4259** | **1291** | **1.20** |  |
|  | **hsa-miR-4785** | **1900** | **1.20** |  |
|  | **hsa-miR-4690-5p** | **1732** | **1.20** |  |
|  | **hsa-miR-3132** | **1153** | **1.20** |  |
|  | **hsa-miR-765** | **508** | **1.18** |  |
|  | **hsa-miR-4703-3p** | **1750** | **1.18** |  |
|  | **hsa-miR-223-5p** | **698** | **1.17** |  |
|  | **hsa-miR-3166** | **1196** | **1.17** |  |
|  | **hsa-miR-516b-5p** | **321** | **1.17** |  |
|  | **hsa-miR-4697-5p** | **1743** | **1.17** |  |
|  | **hsa-miR-5197-3p** | **1427** | **1.17** |  |
|  | **hsa-miR-517b-3p** | **319** | **1.16** |  |
|  | **hsa-miR-1228-3p** | **783** | **1.16** |  |
|  | **hsa-miR-516b-3p** | **322** | **1.16** |  |
|  | **hsa-miR-4461** | **1525** | **1.16** |  |
|  | **hsa-miR-3154** | **1184** | **1.16** |  |
|  | **hsa-miR-4279** | **1329** | **1.16** |  |
|  | **hsa-miR-3126-3p** | **1137** | **1.15** |  |
|  | **hsa-miR-451a** | **244** | **1.15** |  |
|  | **hsa-miR-3939** | **1106** | **1.15** |  |
|  | **hsa-miR-4272** | **1314** | **1.15** |  |
|  | **hsa-miR-1289** | **841** | **1.15** |  |
|  | **hsa-miR-3661** | **1020** | **1.13** |  |
|  | **hsa-miR-485-5p** | **254** | **1.13** |  |
|  | **hsa-miR-1271-5p** | **815** | **1.13** |  |
|  | **hsa-miR-363-3p** | **171** | **1.13** |  |
|  | **hsa-miR-4655-5p** | **1681** | **1.13** |  |
|  | **hsa-miR-92b-5p** | **607** | **1.13** |  |
|  | **hsa-miR-4536-3p** | **1401** | **1.13** |  |
|  | **hsa-miR-4306** | **1269** | **1.12** |  |
|  | **hsa-miR-325** | **223** | **1.11** |  |
|  | **hsa-miR-212-5p** | **1386** | **1.11** |  |
|  | **hsa-miR-520e** | **287** | **1.11** |  |
|  | **hsa-miR-628-5p** | **632** | **1.11** |  |
|  | **hsa-miR-936** | **765** | **1.11** |  |
|  | **hsa-miR-3127-5p** | **1139** | **1.11** |  |
|  | **hsa-miR-3180-3p** | **1214** | **1.11** |  |
|  | **hsa-miR-371b-3p** | **1839** | **1.09** |  |
|  | **hsa-miR-1266** | **890** | **1.09** |  |
|  | **hsa-miR-218-1-3p** | **641** | **1.09** |  |
|  | **hsa-miR-3672** | **1033** | **1.09** |  |
|  | **hsa-miR-181b-3p** | **1382** | **1.08** |  |
|  | **hsa-miR-3149** | **1178** | **1.08** |  |
|  | **hsa-miR-215** | **85** | **1.08** |  |
|  | **hsa-miR-3684** | **1050** | **1.07** |  |
|  | **hsa-miR-4723-5p** | **2015** | **1.07** |  |
|  | **hsa-miR-302a-3p** | **159** | **1.07** |  |
|  | **hsa-miR-1178-5p** | **1394** | **1.07** |  |
|  | **hsa-miR-764** | **1121** | **1.07** |  |
|  | **hsa-miR-520a-3p** | **296** | **1.07** |  |
|  | **hsa-miR-133b** | **222** | **1.06** |  |
|  | **hsa-miR-373-3p** | **193** | **1.05** |  |
|  | **hsa-miR-144-5p** | **558** | **1.05** |  |
|  | **hsa-miR-367-5p** | **659** | **1.05** |  |
|  | **hsa-miR-302d-5p** | **665** | **1.05** |  |
|  | **hsa-miR-3679-3p** | **1043** | **1.04** |  |
|  | **hsa-miR-551b-5p** | **609** | **1.03** |  |
|  | **hsa-miR-5693** | **2098** | **1.03** |  |
|  | **hsa-miR-553** | **368** | **1.03** |  |
|  | **hsa-miR-3200-5p** | **1248** | **1.02** |  |
|  | **hsa-miR-552** | **367** | **1.02** |  |
|  | **hsa-miR-450b-3p** | **725** | **1.02** |  |
|  | **hsa-miR-5004-3p** | **1979** | **1.02** |  |
|  | **hsa-miR-760** | **753** | **1.02** |  |
|  | **hsa-miR-130b-3p** | **166** | **1.01** |  |
|  | **hsa-miR-3676-3p** | **1038** | **1.01** |  |
|  | **hsa-miR-4716-3p** | **1782** | **1.01** |  |
