# Supplementary material for: Screening of microRNAs for a repressor of hepatitis B virus replication
Source: Oncotarget. 2018 Jul 6;9(52):29857–68. doi: 10.18632/oncotarget.25557 (PMC6057454; doi:10.18632/oncotarget.25557)
Supplement: Supplementary file 3 [file oncotarget-09-29857-s003.docx]

**Supplementary Table 2: miRNAs with positive effect of HBV replication in initial screening**

|  | **miRNA** | **miRNA mimic ID of library** | **Reverse_z-score** |  |
| --- | --- | --- | --- | --- |
|  | **hsa-miR-378g** | **1473** | **-4.85** |  |
|  | **hsa-miR-4727-5p** | **1805** | **-4.67** |  |
|  | **hsa-miR-4674** | **1713** | **-4.67** |  |
|  | **hsa-miR-4667-5p** | **2009** | **-4.39** |  |
|  | **hsa-miR-5591-5p** | **2076** | **-4.38** |  |
|  | **hsa-miR-4725-3p** | **1800** | **-4.33** |  |
|  | **hsa-miR-4686** | **1726** | **-4.23** |  |
|  | **hsa-miR-3168** | **1199** | **-4.07** |  |
|  | **hsa-miR-4429** | **1480** | **-3.90** |  |
|  | **hsa-miR-4652-5p** | **1667** | **-3.78** |  |
|  | **hsa-miR-2681-5p** | **1453** | **-3.65** |  |
|  | **hsa-miR-4676-5p** | **1716** | **-3.62** |  |
|  | **hsa-miR-4700-5p** | **1748** | **-3.55** |  |
|  | **hsa-miR-3973** | **1640** | **-3.42** |  |
|  | **hsa-miR-6721-5p** | **2221** | **-3.33** |  |
|  | **hsa-miR-3170** | **1201** | **-3.26** |  |
|  | **hsa-miR-4430** | **1481** | **-3.20** |  |
|  | **hsa-miR-5190** | **1997** | **-3.13** |  |
|  | **hsa-miR-6502-5p** | **2175** | **-3.06** |  |
|  | **hsa-miR-4650-5p** | **1664** | **-3.03** |  |
|  | **hsa-miR-3074-5p** | **2240** | **-2.98** |  |
|  | **hsa-miR-148b-5p** | **586** | **-2.93** |  |
|  | **hsa-miR-548y** | **1105** | **-2.90** |  |
|  | **hsa-miR-3147** | **1175** | **-2.89** |  |
|  | **hsa-miR-4538** | **1635** | **-2.89** |  |
|  | **hsa-miR-548t-5p** | **1165** | **-2.85** |  |
|  | **hsa-miR-1587** | **1632** | **-2.83** |  |
|  | **hsa-miR-4777-3p** | **1888** | **-2.82** |  |
|  | **hsa-miR-154-3p** | **142** | **-2.78** |  |
|  | **hsa-miR-4487** | **1565** | **-2.74** |  |
|  | **hsa-miR-99b-3p** | **574** | **-2.74** |  |
|  | **hsa-miR-6723-5p** | **2224** | **-2.70** |  |
|  | **hsa-miR-3160-3p** | **1190** | **-2.68** |  |
|  | **hsa-miR-3689c** | **1550** | **-2.65** |  |
|  | **hsa-let-7f-2-3p** | **643** | **-2.64** |  |
|  | **hsa-miR-4797-5p** | **1923** | **-2.62** |  |
|  | **hsa-miR-3174** | **1207** | **-2.62** |  |
|  | **hsa-miR-664b-5p** | **2046** | **-2.61** |  |
|  | **hsa-miR-4453** | **1517** | **-2.56** |  |
|  | **hsa-miR-4792** | **1915** | **-2.55** |  |
|  | **hsa-miR-937-5p** | **1355** | **-2.54** |  |
|  | **hsa-miR-4671-3p** | **1710** | **-2.52** |  |
|  | **hsa-miR-3689e** | **1553** | **-2.52** |  |
|  | **hsa-miR-320c** | **812** | **-2.49** |  |
|  | **hsa-miR-4646-5p** | **1661** | **-2.47** |  |
|  | **hsa-miR-3129-3p** | **2233** | **-2.46** |  |
|  | **hsa-miR-548x-5p** | **2254** | **-2.44** |  |
|  | **hsa-miR-548v** | **1176** | **-2.44** |  |
|  | **hsa-miR-892c-3p** | **2226** | **-2.42** |  |
|  | **hsa-miR-3591-5p** | **1828** | **-2.41** |  |
|  | **hsa-miR-3192** | **1231** | **-2.40** |  |
|  | **hsa-miR-604** | **421** | **-2.38** |  |
|  | **hsa-miR-548w** | **1215** | **-2.37** |  |
|  | **hsa-miR-3153** | **1182** | **-2.35** |  |
|  | **hsa-miR-323b-5p** | **1205** | **-2.31** |  |
|  | **hsa-miR-4656** | **1682** | **-2.29** |  |
|  | **hsa-miR-4455** | **1519** | **-2.29** |  |
|  | **hsa-miR-3144-5p** | **1170** | **-2.28** |  |
|  | **hsa-miR-6720-3p** | **2220** | **-2.28** |  |
|  | **hsa-miR-3127-3p** | **2232** | **-2.25** |  |
|  | **hsa-miR-5682** | **2081** | **-2.25** |  |
|  | **hsa-miR-616-3p** | **628** | **-2.23** |  |
|  | **hsa-miR-1250** | **865** | **-2.23** |  |
|  | **hsa-miR-4671-5p** | **1709** | **-2.22** |  |
|  | **hsa-miR-4768-3p** | **1874** | **-2.20** |  |
|  | **hsa-miR-5690** | **2094** | **-2.18** |  |
|  | **hsa-miR-5579-3p** | **2044** | **-2.17** |  |
|  | **hsa-miR-4526** | **1618** | **-2.16** |  |
|  | **hsa-miR-4450** | **1512** | **-2.16** |  |
|  | **hsa-miR-3173-5p** | **2246** | **-2.16** |  |
|  | **hsa-miR-497-5p** | **282** | **-2.14** |  |
|  | **hsa-miR-411-3p** | **636** | **-2.13** |  |
|  | **hsa-miR-3136-3p** | **2234** | **-2.12** |  |
|  | **hsa-miR-9-3p** | **131** | **-2.12** |  |
|  | **hsa-miR-6722-3p** | **2223** | **-2.12** |  |
|  | **hsa-miR-4703-5p** | **1749** | **-2.11** |  |
|  | **hsa-miR-6719-3p** | **2219** | **-2.09** |  |
|  | **hsa-miR-4724-3p** | **1798** | **-2.09** |  |
|  | **hsa-miR-4722-3p** | **1793** | **-2.08** |  |
|  | **hsa-miR-4796-5p** | **1922** | **-2.06** |  |
|  | **hsa-miR-4300** | **1264** | **-2.06** |  |
|  | **hsa-miR-3178** | **1211** | **-2.06** |  |
|  | **hsa-miR-3169** | **1200** | **-2.04** |  |
|  | **hsa-miR-3158-3p** | **1188** | **-2.04** |  |
|  | **hsa-miR-5694** | **2099** | **-2.04** |  |
|  | **hsa-miR-4327** | **1300** | **-2.02** |  |
|  | **hsa-miR-3120-5p** | **2229** | **-2.02** |  |
|  | **hsa-miR-3689f** | **1554** | **-2.00** |  |
|  | **hsa-miR-3148** | **1177** | **-2.00** |  |
|  | **hsa-miR-1301** | **816** | **-1.98** |  |
|  | **hsa-miR-3137** | **1161** | **-1.95** |  |
|  | **hsa-miR-4675** | **1714** | **-1.92** |  |
|  | **hsa-miR-4638-5p** | **1652** | **-1.91** |  |
|  | **hsa-miR-4422** | **1470** | **-1.90** |  |
|  | **hsa-miR-451b** | **1797** | **-1.88** |  |
|  | **hsa-miR-1293** | **845** | **-1.87** |  |
|  | **hsa-miR-3141** | **1166** | **-1.87** |  |
|  | **hsa-miR-4636** | **1650** | **-1.86** |  |
|  | **hsa-miR-3065-5p** | **1221** | **-1.86** |  |
|  | **hsa-miR-4669** | **1706** | **-1.85** |  |
|  | **hsa-miR-5095** | **1946** | **-1.84** |  |
|  | **hsa-miR-4793-5p** | **1916** | **-1.81** |  |
|  | **hsa-miR-4793-3p** | **1917** | **-1.81** |  |
|  | **hsa-miR-3143** | **1168** | **-1.80** |  |
|  | **hsa-miR-4465** | **1534** | **-1.80** |  |
|  | **hsa-miR-2964a-5p** | **1704** | **-1.76** |  |
|  | **hsa-miR-3193** | **1232** | **-1.76** |  |
|  | **hsa-miR-5189** | **1423** | **-1.76** |  |
|  | **hsa-miR-4647** | **1663** | **-1.75** |  |
|  | **hsa-miR-4790-5p** | **1912** | **-1.73** |  |
|  | **hsa-miR-4483** | **1561** | **-1.73** |  |
|  | **hsa-miR-1185-1-3p** | **1366** | **-1.72** |  |
|  | **hsa-miR-204-3p** | **1385** | **-1.72** |  |
|  | **hsa-miR-4665-3p** | **1699** | **-1.72** |  |
|  | **hsa-miR-3162-3p** | **2245** | **-1.71** |  |
|  | **hsa-miR-1323** | **814** | **-1.67** |  |
|  | **hsa-miR-3960** | **1638** | **-1.66** |  |
|  | **hsa-miR-4740-5p** | **1823** | **-1.65** |  |
|  | **hsa-miR-4634** | **1648** | **-1.62** |  |
|  | **hsa-miR-4677-3p** | **1717** | **-1.60** |  |
|  | **hsa-miR-4635** | **1649** | **-1.60** |  |
|  | **hsa-miR-3124-3p** | **2231** | **-1.59** |  |
|  | **hsa-miR-4438** | **1492** | **-1.58** |  |
|  | **hsa-miR-548ab** | **1463** | **-1.57** |  |
|  | **hsa-miR-548av-5p** | **2079** | **-1.56** |  |
|  | **hsa-miR-1245a** | **860** | **-1.54** |  |
|  | **hsa-miR-3194-3p** | **2253** | **-1.54** |  |
|  | **hsa-miR-4721** | **1792** | **-1.54** |  |
|  | **hsa-miR-320d** | **922** | **-1.54** |  |
|  | **hsa-miR-548ay-5p** | **2166** | **-1.54** |  |
|  | **hsa-miR-4486** | **1564** | **-1.53** |  |
|  | **hsa-miR-3065-3p** | **1222** | **-1.52** |  |
|  | **hsa-miR-374a-3p** | **661** | **-1.51** |  |
|  | **hsa-miR-548am-5p** | **1631** | **-1.51** |  |
|  | **hsa-miR-497-3p** | **593** | **-1.51** |  |
|  | **hsa-miR-1185-2-3p** | **1387** | **-1.50** |  |
|  | **hsa-miR-5680** | **2084** | **-1.49** |  |
|  | **hsa-miR-374c-3p** | **1446** | **-1.48** |  |
|  | **hsa-miR-3140-3p** | **1164** | **-1.48** |  |
|  | **hsa-miR-4735-5p** | **1815** | **-1.47** |  |
|  | **hsa-miR-1277-5p** | **1357** | **-1.47** |  |
|  | **hsa-miR-323b-3p** | **1206** | **-1.47** |  |
|  | **hsa-miR-4520b-5p** | **1795** | **-1.46** |  |
|  | **hsa-miR-4421** | **1469** | **-1.45** |  |
|  | **hsa-miR-5583-5p** | **2056** | **-1.45** |  |
|  | **hsa-miR-4680-3p** | **2012** | **-1.45** |  |
|  | **hsa-miR-5687** | **2090** | **-1.44** |  |
|  | **hsa-miR-5007-3p** | **1983** | **-1.44** |  |
|  | **hsa-miR-4491** | **1570** | **-1.44** |  |
|  | **hsa-miR-4645-5p** | **1659** | **-1.43** |  |
|  | **hsa-miR-181c-3p** | **541** | **-1.43** |  |
|  | **hsa-miR-4446-3p** | **1506** | **-1.43** |  |
|  | **hsa-miR-3150a-5p** | **2238** | **-1.43** |  |
|  | **hsa-miR-4451** | **1515** | **-1.42** |  |
|  | **hsa-miR-3150a-3p** | **1179** | **-1.41** |  |
|  | **hsa-miR-3191-3p** | **1230** | **-1.41** |  |
|  | **hsa-miR-3978** | **1645** | **-1.40** |  |
|  | **hsa-miR-501-3p** | **668** | **-1.39** |  |
|  | **hsa-miR-4473** | **1542** | **-1.39** |  |
|  | **hsa-miR-4729** | **1806** | **-1.39** |  |
|  | **hsa-miR-4447** | **1508** | **-1.38** |  |
|  | **hsa-miR-3117-3p** | **1127** | **-1.38** |  |
|  | **hsa-miR-4734** | **1814** | **-1.37** |  |
|  | **hsa-miR-4519** | **1608** | **-1.37** |  |
|  | **hsa-miR-4780** | **1892** | **-1.36** |  |
|  | **hsa-miR-3152-3p** | **1181** | **-1.36** |  |
|  | **hsa-miR-1538** | **931** | **-1.35** |  |
|  | **hsa-miR-3156-3p** | **2241** | **-1.35** |  |
|  | **hsa-miR-5697** | **2102** | **-1.35** |  |
|  | **hsa-miR-26b-3p** | **657** | **-1.35** |  |
|  | **hsa-miR-4682** | **1722** | **-1.35** |  |
|  | **hsa-miR-6084** | **2144** | **-1.34** |  |
|  | **hsa-miR-378i** | **1628** | **-1.34** |  |
|  | **hsa-miR-3974** | **1641** | **-1.33** |  |
|  | **hsa-miR-4528** | **1620** | **-1.33** |  |
|  | **hsa-miR-3157-3p** | **2242** | **-1.32** |  |
|  | **hsa-miR-4480** | **1557** | **-1.32** |  |
|  | **hsa-miR-4750-5p** | **1993** | **-1.32** |  |
|  | **hsa-miR-3187-3p** | **1225** | **-1.32** |  |
|  | **hsa-miR-3182** | **1217** | **-1.31** |  |
|  | **hsa-miR-455-3p** | **673** | **-1.31** |  |
|  | **hsa-miR-4694-3p** | **1739** | **-1.31** |  |
|  | **hsa-miR-3181** | **1216** | **-1.30** |  |
|  | **hsa-miR-4670-5p** | **1707** | **-1.29** |  |
|  | **hsa-miR-4743-3p** | **1966** | **-1.29** |  |
|  | **hsa-miR-5590-5p** | **2074** | **-1.28** |  |
|  | **hsa-miR-3155a** | **1185** | **-1.27** |  |
|  | **hsa-miR-4761-3p** | **1862** | **-1.27** |  |
|  | **hsa-miR-1193** | **1204** | **-1.27** |  |
|  | **hsa-miR-548ay-3p** | **2167** | **-1.26** |  |
|  | **hsa-miR-4482-3p** | **1560** | **-1.26** |  |
|  | **hsa-miR-4659b-3p** | **1693** | **-1.26** |  |
|  | **hsa-miR-3158-5p** | **2243** | **-1.26** |  |
|  | **hsa-miR-4731-5p** | **1808** | **-1.26** |  |
|  | **hsa-miR-1226-3p** | **781** | **-1.26** |  |
|  | **hsa-miR-502-3p** | **666** | **-1.25** |  |
|  | **hsa-miR-6508-5p** | **2186** | **-1.25** |  |
|  | **hsa-miR-6081** | **2141** | **-1.24** |  |
|  | **hsa-miR-548ap-3p** | **1986** | **-1.24** |  |
|  | **hsa-miR-4672** | **1711** | **-1.24** |  |
|  | **hsa-miR-3189-5p** | **2250** | **-1.24** |  |
|  | **hsa-miR-4478** | **1549** | **-1.23** |  |
|  | **hsa-miR-4714-5p** | **1779** | **-1.23** |  |
|  | **hsa-miR-548a-3p** | **401** | **-1.22** |  |
|  | **hsa-miR-4433-5p** | **1486** | **-1.22** |  |
|  | **hsa-miR-4732-3p** | **1811** | **-1.22** |  |
|  | **hsa-miR-1200** | **827** | **-1.21** |  |
|  | **hsa-miR-5589-5p** | **2072** | **-1.20** |  |
|  | **hsa-miR-5590-3p** | **2075** | **-1.20** |  |
|  | **hsa-miR-5588-3p** | **2071** | **-1.20** |  |
|  | **hsa-miR-4756-3p** | **1846** | **-1.20** |  |
|  | **hsa-miR-5089-3p** | **1957** | **-1.19** |  |
|  | **hsa-miR-6500-5p** | **2169** | **-1.19** |  |
|  | **hsa-miR-1207-3p** | **834** | **-1.18** |  |
|  | **hsa-miR-5686** | **2089** | **-1.18** |  |
|  | **hsa-miR-4794** | **1918** | **-1.18** |  |
|  | **hsa-miR-450a-3p** | **1379** | **-1.18** |  |
|  | **hsa-miR-4666a-5p** | **1701** | **-1.17** |  |
|  | **hsa-miR-4687-5p** | **1727** | **-1.16** |  |
|  | **hsa-miR-3156-5p** | **1186** | **-1.16** |  |
|  | **hsa-miR-1343** | **1729** | **-1.15** |  |
|  | **hsa-miR-4477b** | **1548** | **-1.15** |  |
|  | **hsa-miR-5688** | **2091** | **-1.15** |  |
|  | **hsa-miR-5093** | **1960** | **-1.15** |  |
|  | **hsa-miR-571** | **386** | **-1.15** |  |
|  | **hsa-miR-518a-5p** | **598** | **-1.13** |  |
|  | **hsa-miR-548am-3p** | **1630** | **-1.13** |  |
|  | **hsa-miR-6128** | **2155** | **-1.13** |  |
|  | **hsa-miR-6507-5p** | **2185** | **-1.13** |  |
|  | **hsa-miR-330-5p** | **581** | **-1.13** |  |
|  | **hsa-miR-29b-1-5p** | **651** | **-1.13** |  |
|  | **hsa-miR-1306-5p** | **1365** | **-1.12** |  |
|  | **hsa-miR-29a-5p** | **679** | **-1.12** |  |
|  | **hsa-miR-624-3p** | **630** | **-1.12** |  |
|  | **hsa-miR-4431** | **1483** | **-1.12** |  |
|  | **hsa-miR-4668-3p** | **1702** | **-1.12** |  |
|  | **hsa-miR-4417** | **1464** | **-1.11** |  |
|  | **hsa-miR-182-3p** | **73** | **-1.11** |  |
|  | **hsa-miR-4516** | **1605** | **-1.11** |  |
|  | **hsa-miR-548ad** | **1482** | **-1.10** |  |
|  | **hsa-miR-3691-3p** | **1436** | **-1.09** |  |
|  | **hsa-miR-6089** | **2149** | **-1.09** |  |
|  | **hsa-miR-4741** | **1824** | **-1.08** |  |
|  | **hsa-miR-4705** | **1753** | **-1.08** |  |
|  | **hsa-miR-378f** | **1467** | **-1.08** |  |
|  | **hsa-miR-6500-3p** | **2168** | **-1.07** |  |
|  | **hsa-miR-4735-3p** | **1816** | **-1.07** |  |
|  | **hsa-miR-4474-5p** | **1543** | **-1.07** |  |
|  | **hsa-miR-3975** | **1642** | **-1.07** |  |
|  | **hsa-miR-589-3p** | **406** | **-1.06** |  |
|  | **hsa-miR-4495** | **1574** | **-1.04** |  |
|  | **hsa-miR-4732-5p** | **1810** | **-1.04** |  |
|  | **hsa-miR-1184** | **826** | **-1.04** |  |
|  | **hsa-miR-4540** | **1637** | **-1.04** |  |
|  | **hsa-miR-4742-5p** | **1826** | **-1.03** |  |
|  | **hsa-miR-1911-5p** | **938** | **-1.03** |  |
|  | **hsa-miR-4638-3p** | **1653** | **-1.02** |  |
|  | **hsa-miR-4742-3p** | **1825** | **-1.02** |  |
|  | **hsa-miR-138-2-3p** | **554** | **-1.02** |  |
|  | **hsa-miR-548at-3p** | **2053** | **-1.00** |  |
|  |  |  |  |  |
