# Supplementary material for: Screening of microRNAs for a repressor of hepatitis B virus replication
Source: Oncotarget. 2018 Jul 6;9(52):29857–68. doi: 10.18632/oncotarget.25557 (PMC6057454; doi:10.18632/oncotarget.25557)
Supplement: Supplementary file 4 [file oncotarget-09-29857-s004.docx]

**Supplementary Table 3: Putative target genes of 9 candidate miRNAs**

|  |  |  |  |  |
| --- | --- | --- | --- | --- |
|  | **Gene Symbol** | **Gene name** | **Cumulative weighted  context++ score** |  |
|  |  |  |  |  |
|  | **miR-204-5p** |  |  |  |
|  | RAB22A | RAB22A, member RAS oncogene family | -0.77 |  |
|  | DRAP1 | DR1-associated protein 1 (negative cofactor 2 alpha) | -0.71 |  |
|  | PHOX2B | paired-like homeobox 2b | -0.71 |  |
|  | COX5A | cytochrome c oxidase subunit Va | -0.67 |  |
|  | TPT1 | tumor protein, translationally-controlled 1 | -0.65 |  |
|  | MAPRE2 | microtubule-associated protein, RP/EB family, member 2 | -0.64 |  |
|  | FAM160A2 | family with sequence similarity 160, member A2 | -0.63 |  |
|  | C10orf11 | chromosome 10 open reading frame 11 | -0.62 |  |
|  | CAMK1 | calcium/calmodulin-dependent protein kinase I | -0.59 |  |
|  | VWA8 | von Willebrand factor A domain containing 8 | -0.58 |  |
|  |  |  |  |  |
|  | **miR-6133** |  |  |  |
|  | KLK4 | kallikrein-related peptidase 4 | -3.72 |  |
|  | FLJ00104 | HCG1980662; Uncharacterized protein | -3.27 |  |
|  | TET3 | tet methylcytosine dioxygenase 3 | -1.16 |  |
|  | B4GALT3 | UDP-Gal:betaGlcNAc beta 1,4- galactosyltransferase, polypeptide 3 | -0.78 |  |
|  | BCL9L | B-cell CLL/lymphoma 9-like | -0.76 |  |
|  | EIF4EBP1 | eukaryotic translation initiation factor 4E binding protein 1 | -0.75 |  |
|  | SMARCC2 | SWI/SNF related, matrix associated, actin dependent regulator of chromatin,  subfamily c, member 2 | -0.74 |  |
|  | DMBX1 | diencephalon/mesencephalon homeobox 1 | -0.73 |  |
|  | ADAM19 | ADAM metallopeptidase domain 19 | -0.73 |  |
|  | TJP3 | tight junction protein 3 | -0.73 |  |
|  |  |  |  |  |
|  | **miR-519c-3p** |  |  |  |
|  | SLAIN1 | SLAIN motif family, member 1 | -0.84 |  |
|  | BX088651.1 | LOC100126582 protein; Uncharacterized protein | -0.81 |  |
|  | RXFP1 | relaxin/insulin-like family peptide receptor 1 | -0.62 |  |
|  | ZNF277 | zinc finger protein 277 | -0.57 |  |
|  | CMPK1 | cytidine monophosphate (UMP-CMP) kinase 1, cytosolic | -0.52 |  |
|  | COMMD6 | COMM domain containing 6 | -0.51 |  |
|  | ZFP1 | ZFP1 zinc finger protein | -0.50 |  |
|  | ZNF684 | zinc finger protein 684 | -0.50 |  |
|  | GUCY1A3 | guanylate cyclase 1, soluble, alpha 3 | -0.49 |  |
|  | WDR20 | WD repeat domain 20 | -0.48 |  |
|  |  |  |  |  |
|  | **miR-302c-3p** |  |  |  |
|  | C2orf69 | chromosome 2 open reading frame 69 | -1.08 |  |
|  | LMO3 | LIM domain only 3 (rhombotin-like 2) | -0.88 |  |
|  | OLFM3 | olfactomedin 3 | -0.85 |  |
|  | AEBP2 | AE binding protein 2 | -0.80 |  |
|  | KCNJ6 | potassium inwardly-rectifying channel, subfamily J, member 6 | -0.77 |  |
|  | STX11 | syntaxin 11 | -0.76 |  |
|  | UBL3 | ubiquitin-like 3 | -0.75 |  |
|  | MAP3K2 | mitogen-activated protein kinase kinase kinase 2 | -0.73 |  |
|  | LDOC1 | leucine zipper, down-regulated in cancer 1 | -0.71 |  |
|  | KCNJ2 | potassium inwardly-rectifying channel, subfamily J, member 2 | -0.68 |  |
|  |  |  |  |  |
|  | **miR-548c-3p** |  |  |  |
|  | PAK1 | p21 protein (Cdc42/Rac)-activated kinase 1 | -1.00 |  |
|  | PHF21A | PHD finger protein 21A | -1.00 |  |
|  | HSPB11 | heat shock protein family B (small), member 11 | -0.73 |  |
|  | OSGIN2 | oxidative stress induced growth inhibitor family member 2 | -0.65 |  |
|  | SI | sucrase-isomaltase (alpha-glucosidase) | -0.62 |  |
|  | OXSM | 3-oxoacyl-ACP synthase, mitochondrial | -0.49 |  |
|  | KRTAP20-2 | keratin associated protein 20-2 | -0.45 |  |
|  | POLR2H | polymerase (RNA) II (DNA directed) polypeptide H | -0.39 |  |
|  | TEX36 | testis expressed 36 | -0.39 |  |
|  | STT3A | STT3A, subunit of the oligosaccharyltransferase complex (catalytic) | -0.38 |  |
|  |  |  |  |  |
|  | **miR-4633-5p** |  |  |  |
|  | PYROXD1 | pyridine nucleotide-disulphide oxidoreductase domain 1 | -1.16 |  |
|  | GINS4 | GINS complex subunit 4 (Sld5 homolog) | -1.07 |  |
|  | GSAP | gamma-secretase activating protein | -0.93 |  |
|  | TFEC | transcription factor EC | -0.93 |  |
|  | ZBTB37 | zinc finger and BTB domain containing 37 | -0.91 |  |
|  | FAM47E | family with sequence similarity 47, member E | -0.89 |  |
|  | CENPK | centromere protein K | -0.86 |  |
|  | OR12D2 | olfactory receptor, family 12, subfamily D, member 2 | -0.86 |  |
|  | SPG20OS | SPG20 opposite strand | -0.80 |  |
|  | C1orf143 | chromosome 1 open reading frame 143 | -0.80 |  |
|  |  |  |  |  |
|  | **let-7d-5p** |  |  |  |
|  | HMGA2 | high mobility group AT-hook 2 | -2.67 |  |
|  | ARID3B | AT rich interactive domain 3B (BRIGHT-like) | -1.68 |  |
|  | LIN28B | lin-28 homolog B (C. elegans) | -1.58 |  |
|  | FIGN | fidgetin | -1.46 |  |
|  | TRIM71 | tripartite motif containing 71, E3 ubiquitin protein ligase | -1.39 |  |
|  | NR6A1 | nuclear receptor subfamily 6, group A, member 1 | -1.24 |  |
|  | THRSP | thyroid hormone responsive | -1.04 |  |
|  | USP44 | ubiquitin specific peptidase 44 | -1.01 |  |
|  | FAM222B | family with sequence similarity 222, member B | -1.01 |  |
|  | IGDCC3 | immunoglobulin superfamily, DCC subclass, member 3 | -1.01 |  |
|  |  |  |  |  |
|  | **miR-485-5p** |  |  |  |
|  | PTMS | parathymosin | -1.10 |  |
|  | AC007390.5 | HCG1988162; Uncharacterized protein | -0.82 |  |
|  | SUMF2 | sulfatase modifying factor 2 | -0.80 |  |
|  | DNAJC5G | DnaJ (Hsp40) homolog, subfamily C, member 5 gamma | -0.77 |  |
|  | UNC45A | unc-45 homolog A (C. elegans) | -0.75 |  |
|  | SSH3 | slingshot protein phosphatase 3 | -0.69 |  |
|  | RAB8B | RAB8B, member RAS oncogene family | -0.69 |  |
|  | ZBTB39 | zinc finger and BTB domain containing 39 | -0.67 |  |
|  | CKS1B | CDC28 protein kinase regulatory subunit 1B | -0.67 |  |
|  | TPD52L2 | tumor protein D52-like 2 | -0.67 |  |
|  |  |  |  |  |
|  | **miR-2467-3p** |  |  |  |
|  | IL1RN | interleukin 1 receptor antagonist | -1.21 |  |
|  | FLJ00104 | HCG1980662; Uncharacterized protein | -1.17 |  |
|  | ST8SIA2 | ST8 alpha-N-acetyl-neuraminide alpha-2,8-sialyltransferase 2 | -1.10 |  |
|  | SSX3 | synovial sarcoma, X breakpoint 3 | -1.08 |  |
|  | RP5-850E9.3 | Uncharacterized protein | -1.01 |  |
|  | SSX2B | synovial sarcoma, X breakpoint 2B | -1.00 |  |
|  | SSX2 | synovial sarcoma, X breakpoint 2 | -1.00 |  |
|  | PSMD11 | proteasome (prosome, macropain) 26S subunit, non-ATPase, 11 | -1.00 |  |
|  | HIF3A | hypoxia inducible factor 3, alpha subunit | -0.99 |  |
|  | PFDN1 | prefoldin subunit 1 | -0.98 |  |
|  |  |  |  |  |
|  |  |  |  |  |
